# Determination of Quality Indicators for Microvascular Grafts in Cranio-Maxillofacial Surgery—A Retrospective Analysis of 251 Free Flaps

**DOI:** 10.3390/jpm14101061

**Published:** 2024-10-14

**Authors:** Henriette Louise Moellmann, Nadia Karnatz, Ilkan Degirmenci, Majeed Rana

**Affiliations:** 1 Cranio-and-Maxillo Facial Surgery, University Hospital Düsseldorf, Moorenstraße 5, 40225 Düsseldorf, Germany; nadia.karnatz@med.uni-duesseldorf.de (N.K.); rana@med.uni-duesseldorf.de (M.R.); 2 Department of Oral, Maxillofacial and Facial Plastic Surgery, Evangelical Hospital Bethesda, 41061 Mönchengladbach, Germany; ilkan.degirmenci@mg.johanniter-kliniken.de

**Keywords:** microvascular transplant, free flaps, transplant failure, risk assessment

## Abstract

Background: The use of microvascular grafts is the gold standard in oral and maxillofacial surgery for the reconstruction of soft tissue and bony and combined defects. Graft loss is one of the most serious complications in the field of reconstructive surgery. A comprehensive analysis of factors influencing this is, therefore, essential. Methods: This hypothesis-generating study analyzed 251 patient cases of oral and maxillofacial surgery at the University Hospital Düsseldorf from 2016 to 2020 regarding patient- and therapy-specific parameters for their impact on graft survival. Results: Statistically significant influencing factors were found among the 80 parameters examined: treatment with antiplatelet medication and a BMI ≥ 24.5 at the time of surgery had a positive influence on graft survival, while existing diabetes mellitus, atrial fibrillation, tracheostomy, and a longer operation time had a statistically relevant negative influence. Conclusions: This work demonstrates the relevance of patient-specific risk stratification and the need for further research to develop a valid risk profile. Identifying high-risk patients with medium-sized defects, where alternatives to microvascular reconstruction are available, appears to be crucial for the clinical outcome.

## 1. Introduction

Microvascular grafts have been used in oral and maxillofacial surgery for over 50 years now. The first attempts at reconstruction were made in the 1970s by Reuther and Steinau using microvascular anatomized segments of the small intestine for intraoral defect coverage in dogs, which was first used in humans in 1979 [1]. Today, the free forearm graft is a technique that remains the gold standard for the reconstruction of superficial defects. The treatment of 56 patients with this free flap was described for the first time in 1981, describing a graft success rate of 98.3% [2]. The Anterolateral Thigh Flap (ALT-flap) is an option for large-volume soft tissue defects [3]. Bony or combined bony–soft tissue defects are mainly reconstructed using fibula grafts [4,5], osteo-cutaneous scapular flaps [6,7], or microvascular iliac crest grafts [8,9]. Advances in microvascular surgery have made the reconstruction of large and complex defects in the head and neck using free tissue transfer a predictable and individualized approach [10,11]. Despite widely achieved survival rates of over 90%, graft loss remains one of the most feared complications of such procedures. The prevalent etiology of graft loss is venous thrombosis, with arterial thrombosis occurring more frequently at an earlier stage [12,13,14,15]. Furthermore, the choice of donor site and surgical technique also potentially contribute to microvascular thrombosis. However, there is little data on the etiology of many flap failures or the pathophysiologic mechanism of clinically visible tissue loss [16,17,18,19]. There have been attempts to identify factors that have a universal influence on graft survival by means of systematic analyses, but mostly individual operation- or patient-specific factors have been considered [20,21,22,23,24]. Thus, there is a need for more comprehensive analyses of such patient cases, in which patient- and operation-specific factors as well as pre-, peri-, and postoperative therapeutic approaches are considered.

In this retrospective evaluation, we aimed to identify accessible preoperative quality indicators of graft outcome to improve surgical planning, patient education, and postoperative resource allocation and follow-up.

## 2. Materials and Methods

This retrospective study was approved by the local ethics committee at the University of Düsseldorf, Germany (approval number 2021–1290). The study evaluates 251 patient cases from the Department of Oral and Maxillofacial Surgery at the University Hospital Düsseldorf from 2015 to 2020 involving treatment with microvascular grafts. Eighty parameters were evaluated, including patient-specific factors, therapy-specific influences, and peri- and postoperative care factors:

- Patient data (name, age, date of birth, gender, height, weight, and BMI).
- Preoperative (pre-existing conditions that affect the vascular system, etiology of the defect, localization of the defect, and blood parameters).
- Surgery (date, type of graft, resection, ischemia time, duration of surgery, surgical technique, graft, and complications during anastomosis).
- Inpatient stay (complications and length of stay).
- Postoperative course (adjuvant therapy, complications, and blood parameters).

### Statistical Analysis

Each factor was analyzed for its influence on graft survival. Data were analyzed using SPSS (v28, IBM Corp., Armonk, NY, USA) and Jamovi (version 1.6.9, (Computer Software; retrieved from https://www.jamovi.org, accessed on 19 March 2022, Sydney, Australia). The Shapiro–Wilk test was used to determine if the dependent variable was normally distributed, the Levene test was used to determine homoscedasticity, and mean differences were assessed using independent *t*-tests (t) after significant outliers found using boxplots were eliminated. The Mann–Whitney U test (U) or the Yuen test are used to analyze differences in mean values for dependent variables that are not normally distributed or when there is a lack of variance homogeneity. Regarding categorical variables, a contingency table was produced. To examine correlations between categorical variables, the chi-square test was employed. It shows the likelihood that the study’s observations can be applied to the general population. Significance was defined as a *p*-value of less than 0.05, high significance as a value of less than 0.01, and the highest significance as a value less than 0.001. For the hypothesis test, a significance criterion of *p* > 0.05 was established. Binomial logistic regression analysis was used to identify influencing factors that were considered statistically relevant at a significance level of *p* = 0.05.

## 3. Results

**Figure 1:**
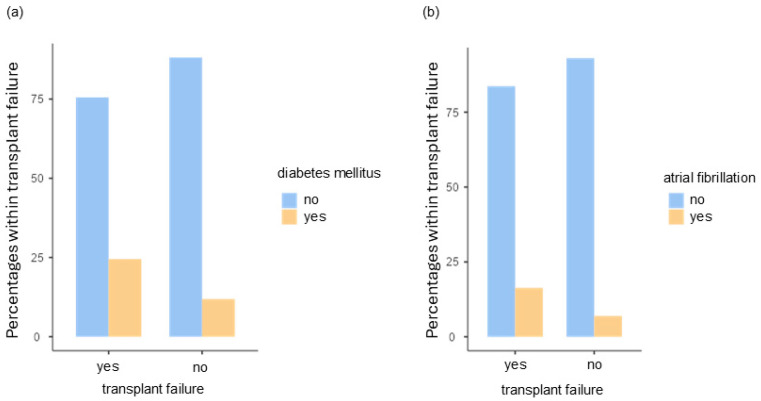
Illustration of patients with (**a**) diabetes and (**b**) atrial fibrillation regarding graft failure.

The present collective consists of a total of 251 transplants, which were transplanted in the Clinic for Oral and Maxillofacial Plastic Surgery during the survey period (January 2015–December 2020). The data of 115 women (45.8%) and 136 men (54.2%) aged 63.0 ± 17.0 resp. 59.4 ± 14.88 years were analyzed. An overview of the data along with the basic parameters is shown in the following table (Table 1):

The correlation between the individual parameters and graft failure was examined using a chi-square test. Risk factors such as alcohol and nicotine consumption showed no significant influence on graft failure in this population, with χ^2^(1) = 0.957, *p* = 0.328, Cramer’s V = 0.072, and χ^2^(1) = 0.180, *p* = 0.672, Cramer’s V = 0.0295. The same applies to the ASA classification with χ^2^(1) = 0.356, *p* = 0.949, Cramer’s V = 0.0378. Here, graft failure occurs primarily in the ASA II and III groups, with frequencies of 50.0% (n = 24) and 37.5% (n = 18), respectively. In group I, the rate of graft failure is 10.4% (n = 5), and in group IV, 2.1% (n = 1). The captured pre-existing conditions that affect the vascular system are shown in Table 2:

Considering the influence of pre-existing disease, the presence of diabetes with χ^2^(1) = 5.10, *p* = 0.024, Cramer’s V = 0.143, shows a significantly higher risk of graft failure. With an OR of 0.416 95%CI [0.191; 0.905], the risk is 0.416-fold higher (see Figure 1a). With χ^2^ (1) = 4.35, *p* = 0.037, Cramer’s V = 0.132, there is also a significant correlation between the incidence of atrial fibrillation and transplant failure. With an OR of OR 0.382 95%CI [0.150; 0.969], the risk of experiencing graft failure is 0.382-fold higher (see Figure 1b).

For the other pre-existing conditions affecting the vascular system, there were no significant correlations for hypercholesterolemia (χ^2^(1) = 2.58, *p* = 0.108, Cramer’s V = 0.101), hypertension (χ^2^(1) = 0.598, *p* = 0.439, Cramer’s V = 0.0488), coronary heart disease χ^2^(1) = 3.22, *p* = 0.073, Cramer’s V = 0.113), arteriosclerosis (χ^2^(1) = 0.0732, *p* = 0.787, Cramer’s V = 0.0171), or post-thromboembolic events (χ^2^(1) = 1.21, *p* = 0.272, Cramer’s V = 0.0694). Reconstruction using a microvascular graft was performed in 172 cases (68.5%) due to squamous cell carcinoma; in 52 of the cases (20.7%), a secondary reconstruction was performed, and in 17 cases (6.8%), osteoradionecrosis was diagnosed. An overview of other underlying diseases, resection sites, etc., is shown in Table 3.

With χ^2^(1) = 3.46, *p* = 0.063, Cramer’s V = 0.117, there is no significant correlation between the presence of tumor disease and graft failure. The influence of the T-stage on the success of the transplant is not significant in this cohort, with χ^2^(6) = 6.30, *p* = 0.390, Cramer’s V = 0.158. Other factors, such as the site of resection and the associated defect localization, also show no significant impact on graft failure (χ^2^(8) = 9.39, *p* = 0.3109, Cramer’s V = 0.193 and χ^2^(5) = 2.22, *p* = 0.818, Cramer’s V = 0.0940). Fascio-cutaneous grafts (radialis-graft) were the most commonly used graft during surgery, accounting for 157 of the cases (62.5%), followed by osteo-musculocutaneous grafts, such as fibula or scapula flaps in 61 cases (24.3%). Musculo-cutaneous transplants, such as ALT and latissimus dorsi flaps, were used in 31 cases (13.1%). With χ^2^(2) = 5.00, *p* = 0.082, Cramer’s V = 0.141, there is no significant correlation between the choice of graft and graft failure. The anastomosis was performed end-to-end in 98.8% (n = 248) of the arteries and 80.5% (n = 202) of the veins. Further operation-specific parameters are listed in Table 4.

**Figure 2:**
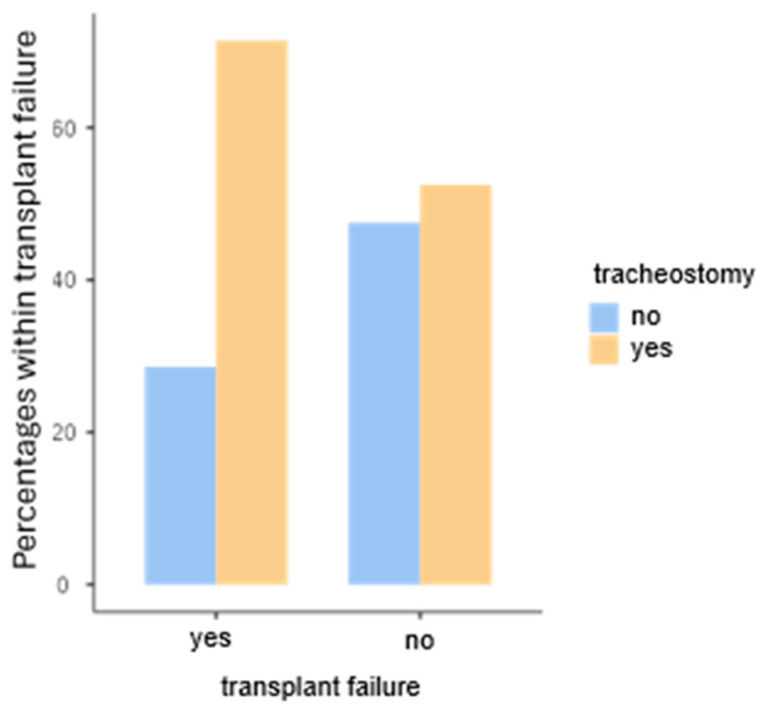
Illustration of the tracheostomized patients regarding transplant failure.

If a percutaneous gastric tube is placed as part of the preoperative preparation, no significant influence on the transplant is observed (χ^2^(1) = 0.805, *p* = 0.369, Cramer’s V = 0.0566). However, if the patient is tracheotomized, there is a significant influence on graft failure (χ^2^(1) = 5.75, *p* = 0.016, Cramer’s V = 0.151). The risk is increased 2.26-fold with an OR of OR 2.26 95%CI [1.15; 4.46] (see Figure 2).

**Figure 3:**
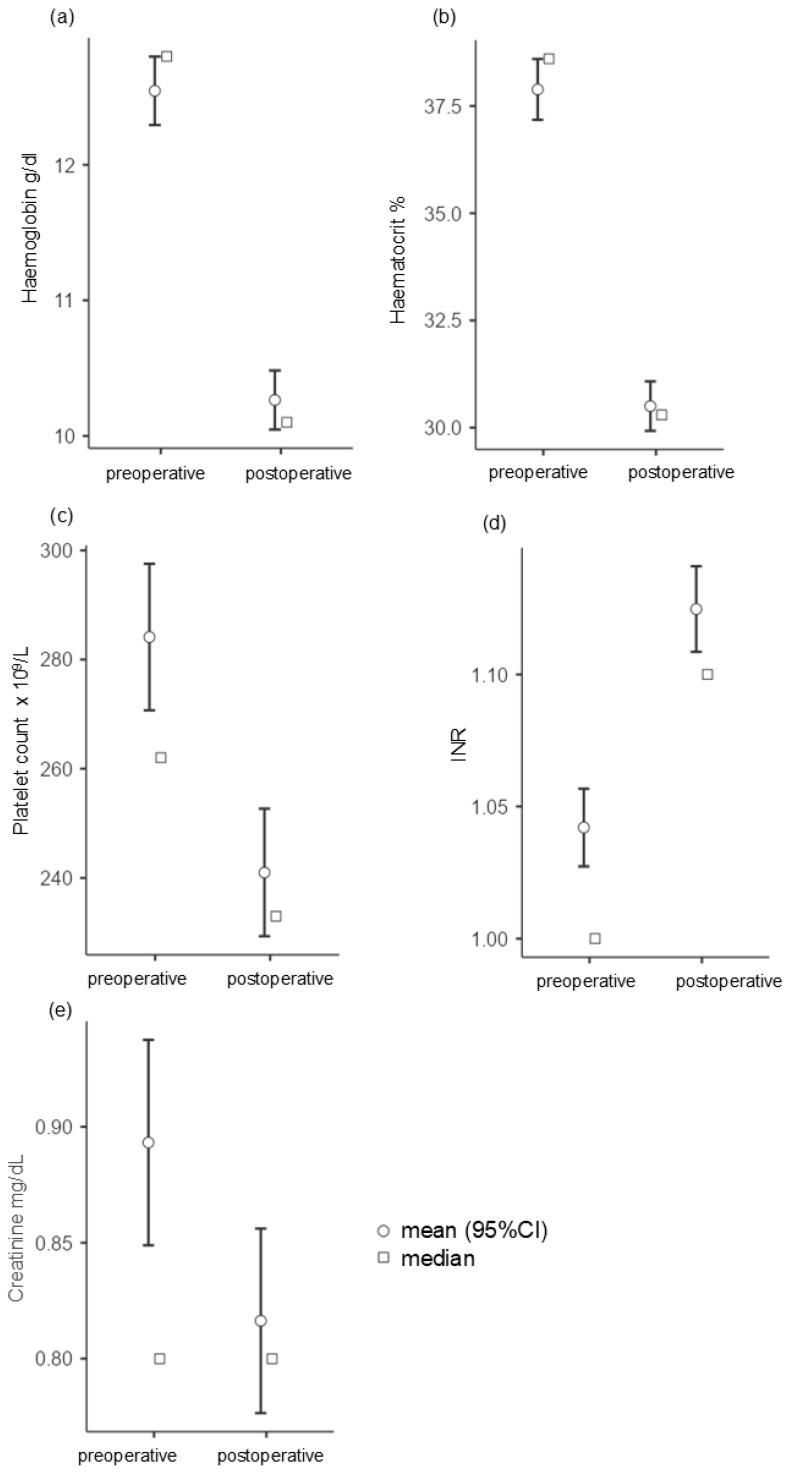
Illustration of the pre- and postoperative values of (**a**) hemoglobin, (**b**) hematocrit, (**c**) platelet count, (**d**) INR. and (**e**) creatinine.

If neck dissection is necessary, there is no significantly higher rate of graft failure (χ^2^(1) = 1.46, *p* = 0.227, Cramer’s V = 0.0762). We also analyzed certain blood values before and after the operation. An overview is shown in the following Table 5:

A comparison of the pre- and postoperative blood values shows a significant decrease in hemoglobin (W = 29015.00, *p* < 0.001, rrb = 0.91), hematocrit (t(250) = 22.50, *p* < 0.001, d = 1.426), platelet count (W = 25005.00, *p* < 0.001, rrb = 0.633), and creatinine (W = 12601.00, *p* < 0.001, rrb = 0.600). The INR increases significantly in the postoperative course (W = 1193.00, *p* < 0.001, rrb = −0.834) (see Figure 3a–e).

A binominal logistic regression was performed to examine whether there was a correlation between the preoperative blood values and graft failure. The binomial logistic regression model was not statistically significant, χ^2^ (5) = 9.58; *p* = 0.088, resulting in a small amount of explained variance [25], as shown by Nagelkerke’s R^2^ = 0.0610. The overall percentage of accuracy in classification was 81.5%, with a sensitivity of 100.0% considering transplant failure and a specificity of 2.13%. With a coefficient of determination of R^2^ = 0.061, a sample size of 248, and a significance level of α = 0.05, the statistical power of five predictors would be 1 − β = 0.88455. The statistical power indicates the probability of committing an error of the 2nd kind. Here, the probability of committing a 2nd type of error would be 11.55%. In 11.55% of cases, the test would not indicate significance, even if it were actually significant [26]. No factor showed a statistically relevant influence with hemoglobin (*p* = 0.441), hematocrit (*p* = 0.230), INR (*p* = 0.281), platelet count (*p* = 0.350), and creatinine (*p* = 0.139). The aim of this study was to identify quality indicators for microvascular transplants. The main focus lies in avoiding microvascular complications. In 31.1% of the cases (n = 78), a thrombosis occurred in the pedicle. This resulted in ischemia in 14.3% (n = 36) and venous congestion of the graft in 16.3% (n = 41). In 69.3% of cases (n = 174), the graft did not show any vascular-associated complications. In 50 cases (19.9%), the anastomosis was successfully revised, and in 49 cases (19.5%), the graft had to be removed. An overview of the vascular-associated local and general complications is shown in Table 6.

A binomial logistic regression was performed to investigate the influence of age, BMI, gender, and other selected parameters on graft failure. The binomial logistic regression model was statistically significant, χ^2^(6) = 26.2; *p* < 0.001, resulting in a low proportion of explained variance [27], as shown by Nagelkerke’s R^2^ = 0.162. The overall percentage accuracy of the classification was 81.6%, with a sensitivity of 14.6% for graft failure and a specificity of 98.0%. With a coefficient of determination of R^2^ = 0.162, a sample size of 244, and a significance level of α = 0.05, one would have a statistical power of 1 − β = 0.99991 with six predictors. The statistical power indicates the probability of committing an error of the 2nd kind. Here, the probability of committing an error of the 2nd kind would be 0.01%. In 0.01% of cases, the test would not indicate significance, even if it were actually significant [26].

Four factors were found to be statistically relevant influencing factors:

1. Higher BMI (median = 24.5; SD = 4.68) as a positively influencing variable (*p*= 0.009; OR = 0.896 95%CI [0.8249; 0.973]);
2. The presence of diabetes mellitus as a negative predictive value (*p* = 0.002; OR = 4.234, 95%CI [1.6858; 10.634]);
3. Long-term medication by means of platelet aggregation inhibition as a positive influencing factor (*p* = 0.041, OR 0.277 95%CI [0.0809; 0.947]);
4. Extension of the operation time increases the probability of graft failure (*p* = 0.011; OR = 1.003, 95% CI [1.0006; 1.005]) (s. Table 7).

## 4. Discussion

As graft loss is one of the most serious complications in microvascular surgery, many attempts have been made to identify factors that jeopardize graft survival. Factors such as surgical experience, careful patient selection, patient-related characteristics, and postoperative care are discussed. Nevertheless, the success and loss of flap surgery is a combination of these factors rather than being determined by a single factor [28,29,30,31,32,33]. Despite many years of experience and successful use of microvascular grafts, the identification of patients at risk of graft failure preoperatively remains challenging [11,16,19,34]. Concretely, the rate of graft failure in microsurgical head and neck reconstruction is reported in the literature as 6.2–9.9% [35,36], with an increased loss rate of 19.9% (49 of 251) in this population.

In an analysis of 565 free flaps, Lese et al. (2021) identified possible risk factors that could lead to flap failure and/or vascular compromise. The patients were divided into three classes: low, medium, and high risk. The classification is based on the etiology of the defect and the presence of coronary artery disease, diabetes, smoking, peripheral arterial occlusive disease, and arterial hypertension. Patients with a moderate risk index were 9.3 times more likely to develop vascular damage than the low-risk group, while patients with a high risk index were 18.6 times more likely (*p* = 0.001) [36]. In our group, classic vascular risk factors, such as nicotine and alcohol consumption or pre-existing conditions like hypertension and coronary artery disease, were not significant. Nevertheless, it is essential to consider and evaluate vascular risk factors in a targeted assessment in order to provide patients with individualized treatment with adjustments to the reconstruction plan or an alternative reconstruction strategy [36].

The influence of the presence of diabetes mellitus on graft survival found in this analysis is in line with the status of the meta-analysis by Caputo et al. (2020) [37]. Vascular changes and the resulting disruption of microcirculation due to permanently elevated blood glucose levels have been extensively researched and relate systemically to the entire vascular system and not just to the specific issues of head and neck surgery [38,39,40]. Kantar et al. (2019) examined a collective of 6030 patients and found evidence that diabetes is not associated with a significantly increased rate of flap failure. However, diabetic patients in particular showed a significantly higher rate of wound complications (e.g., wound dehiscence) [41].

In addition to diabetes mellitus, the results show a significant correlation between the presence of atrial fibrillation and graft failure. In a retrospective analysis of 320 patients undergoing oral cavity composite resection with free flap reconstruction, Ye et al. (2024) showed that at least the general complication rate is increased in patients with atrial fibrillation (OR 2.94; 1.17–7.39) [42]. Further studies have also shown that the recording of atrial fibrillation as part of the preoperative risk assessment is also of major relevance in non-cardiac surgery [43,44]. Similarly to Stevens et al. (2023), our results could not demonstrate a significant influence of flap types on graft failure. The authors compared fascio-cutaneous, osteo-cutaneous, anterolateral thigh, rectus abdominus, latissimus dorsi, fibula, and scapula flaps [19]. The present study demonstrates a correlation between a tracheostomy and graft failure. Poisson et al. (2019) showed in a multicentric study including 215 patients treated by tumor resection with immediate free flap reconstruction for an OSCC that tracheostomy increases the incidence of major surgical complications and delays oral nutrition in the postoperative period [45]. For preventing not only flap-specific but general complications, a strict indication for tracheotomy seems to be advisable. A possible score to predict the need for a tracheostomy [46] or an alternative postoperative procedure with prolonged intubation [47] is discussed in the literature.

After microvascular reconstruction, patients often develop anemia due to iatrogenic hemodilution and acute blood loss. There are some large clinical studies examining the effects of preoperative anemia on transplant failure [48,49]. Hill et al. (2012) showed in an analysis of 156 free flap operations in 147 patients that Hb and Hct were significant predictors of flap failure (*p* < 0.005) and vascular thrombosis (*p* < 0.05) [48]. Our analysis revealed a significant drop in hemoglobin and hematocrit during surgery but not their significance as a predictor of transplant failure. However, these values should be part of the preoperative risk assessment.

The relationship between BMI and the success of a microvascular transplant has already been analyzed, particularly in the context of reconstructive breast surgery. Existing meta-analyses describe a higher incidence of postoperative flap complications and flap failure with an increased BMI [50]. However, the limitations of these evaluations in the field of breast surgery are also highlighted. With regard to head and neck surgery, the data situation is heterogeneous, with no statistically relevant correlations between increased BMI and graft failure [51,52,53]. In 239 head and neck cancer patients, Yu et al. (2024) showed that 38 (15.9%) patients had postoperative complications related to free flap reconstruction. In a multivariate analysis, they found evidence that low BMI (*p* < 0.001), high postoperative CRP level (*p* = 0.005), low hemoglobin level (*p* = 0.012), and inadequate fluid intake (*p* < 0.05) were also independent risk factors for complications [49]. Malnutrition with consecutive hypalbuminemia is widely regarded as a negative predictive value in microvascular reconstructions [54,55,56], although the results obtained in this study show a positive predictive value for graft survival with increased BMI. An additional correlation between BMI and blood albumin levels in relation to graft survival should be determined for further analysis.

The use of platelet aggregation inhibitors in microvascular surgery is controversially debated. The focus lies in perioperative administration, in the sense of prophylactic administration, to prevent vascular occlusion in the area of the graft [57,58,59]. It is generally agreed that further blood-thinning medication beyond the administration of low-molecular-weight heparins for thrombosis prophylaxis has no relevant influence on graft survival. Nevertheless, the influence of preoperative medication with antiplatelet agents is rarely examined, even though studies were unable to show a significant influence [59]. Stevens et al. (2023) showed that platelet-induced hypercoagulability may be a possible cause of some microvascular anastomotic thromboses. Thus, elevated platelet levels could serve as a pathophysiologic surrogate for systemic hypercoagulability. In the cohort of Stevens et al. (2023), patients with preoperative thrombocytosis were found to have an increased risk of early flap failure (OR, 2.67) [19]. This is consistent with our findings that the use of antiplatelet drugs appears to have a protective effect. We were unable to demonstrate a significant association between thrombocytosis and flap failure in our study. Nevertheless, platelets and their inhibition should be considered in risk stratification, as these factors not only have a direct influence on grafting but also on general outcomes. Tarabishy et al. (2020) showed that patients with thrombocytosis undergoing microvascular free flaps are at increased risk for complications, including the need for a blood transfusion, prolonged hospital stays, and reoperation [60].

The analysis shows a direct correlation between the duration of surgery and anesthesia and graft survival [61,62,63]. In addition to the technical skill and experience of the surgeon, organizational factors, such as the pre- and postoperative treatment of the patient in the operating theatre [64] or the composition of the surgical team [65], were also found to have an influence. Thus, due to the urgency of reducing the duration of surgery to increase graft survival, several starting points can be found, which should be integrated into everyday clinical practice.

Shinde et al. (2021) showed a 90-day mortality of 3.2% in 33,845 patients (median age was 63 years) with oral cavity cancer (OCC) [66]. Further studies showed a 90-day mortality rate of less than 3.8% in patients with head and neck cancer [67,68]. In older patients (≥80 years) with T3–4 stage and patients (<80 years) with pre-existing diseases and T3–4, N2–3 stage, 90-day mortality was >10% [66]. For patients (<80 years) with T3–4, N2–3 stage, and patients (<80 years) with T3–4 stage with CD score 1–3, 90-day mortality was 5–10%. In their analysis, older age, more pre-existing conditions, non-private insurance, lower income, treatment in an academic institution, high T and N stages, radical excision, and the presence of positive margins are mentioned as risk factors [66]. At 7.9% with a comparable cohort (age, T-stage, etc.), a similar 90-day mortality is present in our presented study. Shinde et al. (2021) report that 20% of all OCC patients have a medium to high risk of dying within the first 90 days after surgery according to their analysis [66]. This, again, underlines the importance of risk-adapted preoperative assessment of each individual patient and a strict indication for surgical treatment.

Since this study was conducted using retrospective medical records and errors in the electronic medical records may have caused the variables to be overestimated or underestimated, it is inherently limited. Regardless of magnitude, the majority of variables were recorded as binary data to prevent bias and misunderstanding. Further large-scale studies should examine the suitability of the identified risk factors.

This study identified diabetes mellitus, atrial fibrillation, and long operating times as risk factors for graft failure, whereas an elevated BMI and the administration of antiplatelet agents had a protective effect. The work demonstrates the relevance of patient-specific risk stratification along with further research for developing a dedicated risk profile. Foremost, the results need to be taken into account in surgical decision making to improve patient outcomes. The identification of high-risk patients with medium-sized defects, where alternatives to microvascular reconstruction are available, seems to be especially vital.

## 5. Conclusions

Four statistically relevant factors influencing graft survival in microvascular reconstructions of the head and neck region were identified. These findings largely reflect existing scientific knowledge in the field of microvascular reconstruction and aid in deriving concrete measures such as risk stratification, patient selection, and indication for surgery. The development of a preoperative patient-specific risk stratification for treatment with microvascular grafts is highly advisable. Identifying high-risk patients with medium-sized defects, where alternatives to microvascular reconstruction are applicable, appears to be crucial for the clinical outcome.

## Figures and Tables

**Table 1:** Depiction of the data, including basic parameters.

Parameters		n, MD ± SD
Gender	Female	115 (45.8%)
	Male	136 (54.2%)
Age	Female	63.0 ± 17.0 years
	Male	59.4 ± 14.8 years
Height	Female	165.0 ±6.9
	Male	178.0 ± 7.91
Weight	Female	64.4 ± 12.8
	Male	79.9 ± 16.9
BMI	Female	23.9 ± 4.6
	Male	25.2 ± 47
ASA Classification		
	I	32 (12.8%)
	II	123 (49.2%)
	III	89 (35.6%)
	IV	6 (2.4%)
Nicotine abuse		
	NA	44 (17.5%)
	Never	87 (34.7%)
	Former smoker	42 (16.7%)
	Active smoker	78 (31.1%)
	PY	19.4 ± 22.7 (0–104 PY)
Alcohol abuse		
	NA	68 (27.1%)
	Never	109 (43.4%)
	Rarely	2 (0.8%)
	Occasionally	13 (5.2%)
	Daily	40 (15.9%)
	Sober	19 (7.6%)

**Table 2:** Overview of the pre-existing conditions.

Pre-Existing Conditions		n, Percentage (5%)
Hypercholesterolemia	Yes	26 (10.4%)
	No	225 (89.6%)
Diabetes mellitus	Yes	36 (14.3%)
	No	215 (85.7%)
Hypertension	Yes	120 (47.8%)
	No	131 (52.2%)
Coronary heart disease	Yes	24 (9.6%)
	No	227 (90.4%)
Atrial fibrillation, cardiac arrhythmia	Yes	22 (8.8%)
	No	229 (90.4%)
Arteriosclerosis	Yes	23 (9.2%)
	No	228 (90.8%)
Post-thromboembolic events (e.g., LAE)	Yes	34 (13.5%)
	No	217 (86.5%)

**Table 3:** Overview of the underlying disease, site of resection, localization of the defect, and T-Stadium.

Parameters		n, Percentage (%)
Underlying disease	MONJ	3 (1.2%)
	Osteomyelitis	3 (1.2%)
	Osteoradionecrosis	17 (6.8%)
	SCC	172 (68.5%)
	Reconstruction	52 (20.7%)
	Others	4 (1.6%)
Site of resection		
	Lip	2 (0.8%)
	Floor of mouth	6 (2.4%)
	Upper Jaw	33 (13.1%)
	Lower Jaw	67 (26.7%)
	Cranium	16 (6.4%)
	Cheek	21 (8.4%)
	Tongue	27 (10.8%)
	Overlapping	78 (31.1%)
Localization of the defect		
	Left	87 (34.7%)
	Left-accentuated, crossing the midline	9 (3.6%)
	Overlapping	9 (3.6%)
	Mid	36 (14.3%)
	Right	102 (40.6%)
	Right-accentuated, crossing the midline	8 (3.2%)
T-Stadium (n = 172)		
	NA	81 (32.3%)
	Tx	14 (5.6%)
	pT1	41 (16.3%)
	pT2	38 (15.1%)
	pT3	32 (12.7%)
	pT4a	43 (17.1%)
	pT4b	2 (0.8%)

**Table 4:** Depiction of the surgery-related parameters.

Parameters		n, Percentage (%)
Flap design		
	Fascio-cutaneous	157 (62.5%)
	Musculo-cutaneous	33 (13.1%)
	Osteo-musculocutaneous	61 (24.3%)
Arterial anastomosis	End-to-end	248 (98.8%)
	End-to-Side	3 (1.2%)
Venous anastomosis	End-to-end	202 (80.5%)
	End-to-Side	49 (19.5%)
Percutaneous gastric tube	Yes	129 (51.4%)
	No	122 (48.6%)
	Duration (n = 102)	54.9 ± 52.9 (2–120) days
Neck dissection		
Right side	None	128 (51.0%)
	Lymphnode-Extirpation	3 (1.2%)
	Level I–III	94 (37.5%)
	Level I–V	26 (10.4%)
Left side	None	123 (49.0%)
	Lymphnode-Extirpation	4 (1.6%)
	Level I–III	92 (36.7%)
	Level I–V	32 (12.7%)
Duration of surgery		551 ± 170 min
Duration invasive ventilation		37.4 ± 33.3
Length of stay		37.4 ± 33.3 days
Adjuvant therapy		
	None	150 (59.8%)
	Radiation	55 (21.0%
	Radio-Chemotherapy	46 (18.3%)

**Table 5:** Overview of the pre- and postoperative blood values.

Blood Values		MD ± SD
Hemoglobin	Preoperative	12.5 ± 2.04
	Postoperative	10.3 ± 1.76
Hematocrit	Preoperative	37.9 ± 5.73
	Postoperative	30.5 ± 4.65
INR	Preoperative	1.04 ± 0.114
	Postoperative	1.12 ± 0.127
Platelet count	Preoperative	284 ± 109
	Postoperative	241 ± 94.3
Creatinine	Preoperative	0.895 ± 0.357
	Postoperative	0.816 ± 0.321

**Table 6:** Overview of the vascular-associated local and general complications.

Complications		n, Percentage (%)
Thrombosis of the pedicle	Yes	78 (31.1%)
	No	173 (68.9%)
Complications of the flap		
	None	174 (69.3%)
	Ischaemia	36 (14.3%)
	Venous stasis	41 (16.3%)
Other local complications	None	168 (66.9%)
	Bleeding	15 (6%)
	Dehiscence	38 (15.1%)
	Necrosis	18 (7.2%)
	Other	1 (0.4%)
Flap revision without explantation	Yes	50 (19.9%)
	No	201 (80.1%)
Failure of the flap	Yes	49 (19.5%)
	No	202 (80.5%)
General medical complications	None	122 (48.6)
	Yes	51.39%
	Ileus	2
	Peritonitis	3
	Chylous fistula	2
	Pneumonia	14
	Respiratory insufficiency	3
	Pneumogenic sepsis	1
	ARDS (Acute Respiratory Distress Syndrome)	2
	Pneumothorax	3
	Pulmonary embolism	2
	Thrombosis (e.g., DVT, deep vein thrombosis)	4
	Stroke	2
	Cardiac decompensation	2
	Myocardial infarction	5
	Cardiogenic shock	1
	Hypoxia	2
	Cardiopulmonary resuscitation	6
	Multi-organ failure	1
	Acute renal failure	9
	Sepsis	3
	MRSA	10
	3MRGN	1
	Delirium	24
	Others	15
Deceased within 90 days	Yes	19 (7.6%)
	No	232 (92.4%)

**Table 7:** Overview of model coefficients: models for predictability of the preoperative parameters (age, BMI, gender, diabetes mellitus, inhibition of platelet aggregation, and time of surgery) of transplant failure. Note. The cut-off value is set to 0.5.

Model Fit Measures							
					Overall Model Test		
Model	Deviance	AIC	R2McF	R2N	χ2	df	*p*
1	216	230	254	0.162	26.2	6	<0.001
Model Coefficients—Transplant failure yes/no							
						95% Confidence Interval	
Predictor	Estimate	SE	Z	*p*	Odds Ratio	Lower	Upper
Intercept	−0.658	0.775	−0.848	0.396	0.518	0.113	2.37
Age	0.199	0.217	0.914	0.361	1.22	0.797	1.87
BMI	0.302	0.162	1.863	0.062	1.353	0.984	1.86
Gender							
female–male	0.15685	0.35621	0.4403	0.660	1.170	0.5820	2.351
Diabetes mellitus:							
yes/no	144.316	0.46987	30.714	0.002	4.234	16.858	10.634
Inhibition of platelet aggregation:							
yes/no	−128.415	0.62755	−20.463	0.041	0.277	0.0809	0.947
Time of surgery (min)	0.00255	0.00100	25.430	0.011	1.003	10.006	1.005

Note: Estimates represent the log odds of “Transplant failure = no” vs. “Transplant failure = yes”.

## Data Availability

The data presented in this study are available on request from the corresponding author.
